# Design, synthesis and evaluation of a series of potential prodrugs of a Bruton’s tyrosine kinase (BTK) inhibitor

**DOI:** 10.3389/fphar.2023.1162216

**Published:** 2023-03-08

**Authors:** Zhou-Peng Xiao, Min Liao, Xue-Juan Huang, Yu-Tong Wang, Xiao-Cui Lan, Xue-Ying Wang, Xi-Tao Li

**Affiliations:** ^1^ School of Pharmaceutical Sciences (Shenzhen), Sun Yat-sen University, Shenzhen, China; ^2^ BayRay Innovative Center, Shenzhen Bay Laboratory, Shenzhen, China

**Keywords:** prodrug, BTK-bruton’s tyrosine kinase, inhibitor, solubility, DFG-out

## Abstract

BTK has become a particularly attractive therapeutic target in autoimmune diseases and B-cell malignancies, making BTK inhibitors a valuable and important therapeutic option. We present the design, synthesis, and evaluation of a series of prodrugs of a BTK inhibitor with an insoluble 2,5-diaminopyrimidine structure. Tails containing different solubilizing groups were added to the parent molecule *via* an ester linkage. Prodrug **5a** showed good aqueous solubility and could be efficiently converted to the parent in a human plasma stability study. The rational prodrug design was supported by molecular studies and a dramatically reduced BTK kinase-inhibitory potential. Taken together, the chemical, biological, and molecular studies suggest that prodrug derivatization of the 2,5-diaminopyrimidine scaffold could be a potential strategy for advancing this series of BTK inhibitors into the therapeutic arena.

## 1 Introduction

**FIGURE 1:**
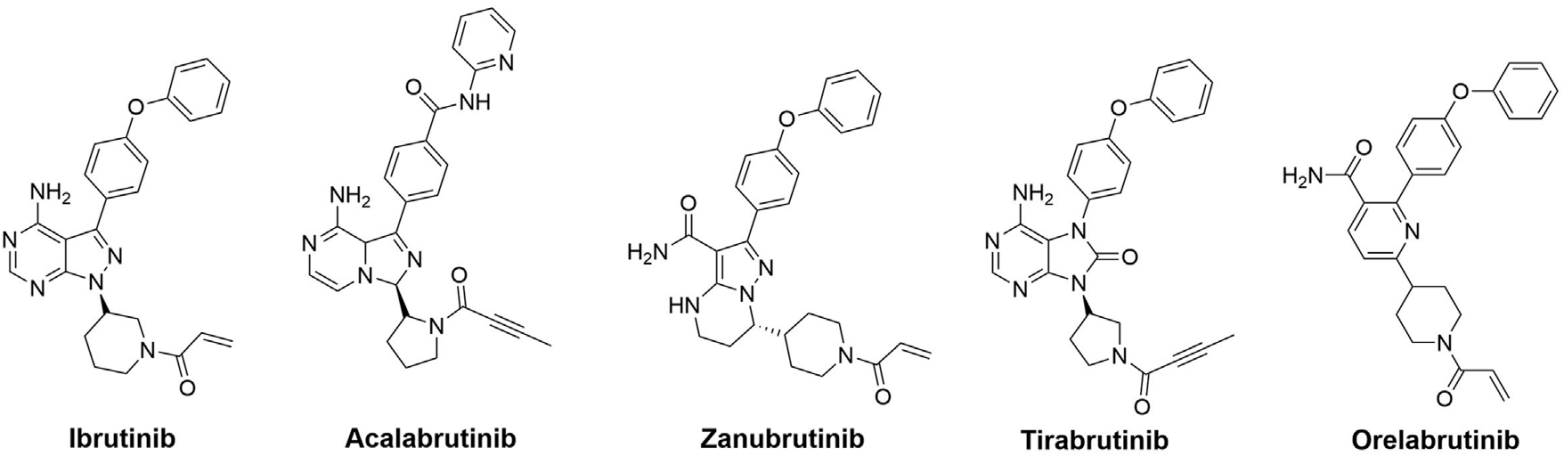
Representative BTK inhibitors on market.

Bruton’s tyrosine kinase (BTK), a non-receptor cytoplasmic kinase of the TEC family, has expression in all hematopoietic cells except T lymphocytes and plasma cells (Brunner et al., 2005; Koprulu and Ellmeier, 2009). BTK is an important component of the BCR signaling system, which is required for B-cell activation, survival, and differentiation (Satterthwaite and Witte, 2000). Overexpression and inappropriate activation of BTK have been implicated in the pathogenesis of several hematologic malignancies (Pal Singh et al., 2018; Burger, 2019). As a result, the pharmacological suppression of BTK is of great therapeutic benefit not only in B-cell, but in other hematological malignancies as well (Rushworth et al., 2014; Li et al., 2021). There has been a significant development of BTK inhibitors over the past decade. Approved drugs include first- and second-generation covalent inhibitors (Figure 1), such as ibrutinib (2013), acalabrutinib (2017), zanubrutinib (2019), tirabrutinib (2020), and orelabrutinib (2020) (George et al., 2020; Ran et al., 2021). Recently, third-generation non-covalent BTK inhibitors have been shown to have potent inhibitory activity against both BTK and the BTK^C481S^ mutant, as well as the capability of overcoming the acquired resistance to covalent inhibitors that is caused by the BTK^C481S^ mutation (Ondrisova and Mraz, 2020). Despite this, new BTK inhibitors with diverse binding mechanisms are urgently needed to be developed in order to overcome unexpected acquired BTK mutations.

**FIGURE 2:**
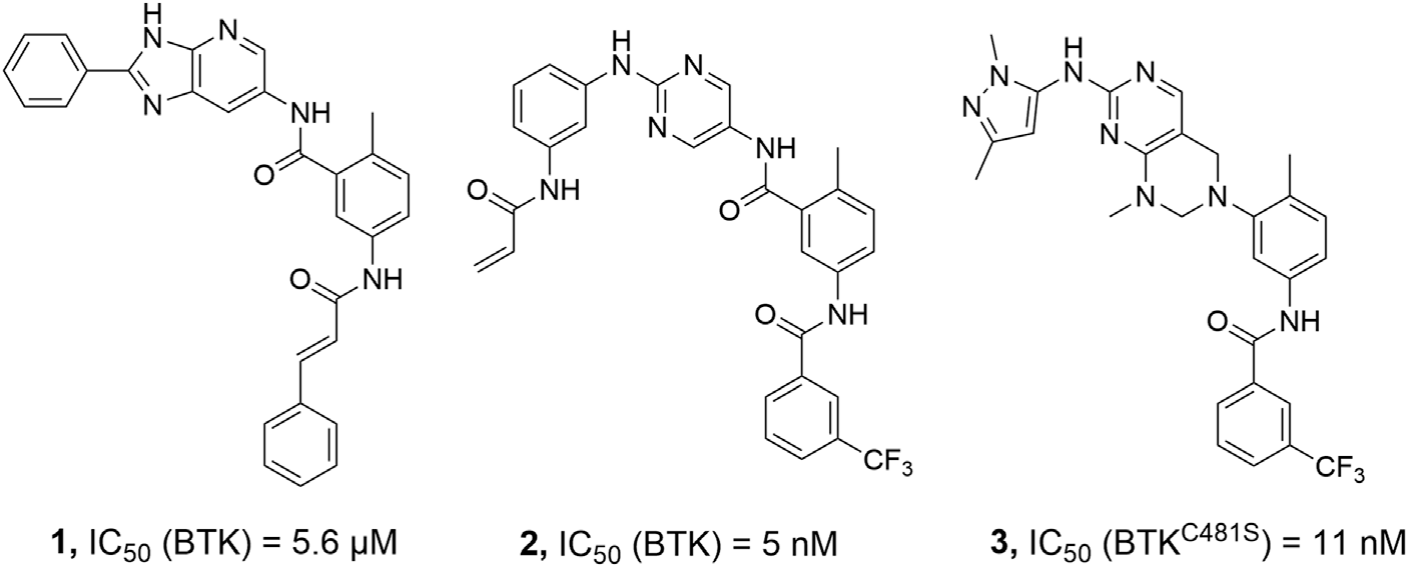
Representative BTK inhibitors with type II structure.

Non-covalent kinase inhibitors are categorized into type-I and type-II on the basis of the kinase conformation they target (Liu and Gray, 2006). Type-II kinase inhibitors are compounds that bind to their target kinases and trap them in an inactive conformation known as DFG-out, by occupying a hydrophobic pocket close to the ATP binding site. These inhibitors are frequently more selective than those targeting the active DFG-in conformations of kinases (Kufareva and Abagyan, 2008). To date, only a few BTK inhibitors have been reported to bind in the DFG-out conformation (Figure 2) (Kuglstatter et al., 2011; Gui et al., 2019). We reported in 2014 that a range of 2,5-diaminopyrimidine covalent BTK inhibitors with a type-II scaffold had effective antiproliferative activity in multiple B-cell lymphoma cell lines as well as significant efficacy in a human tumor xenograft model (Li et al., 2014). Unfortunately, lead compound **2** suffered from poor solubility, only 7.02 μM in FaSSIF (Fasted State Simulated Intestinal Fluid, pH 6.5) medium, and low bioavailability (F% = 0.9%) (Li et al., 2014). As a result, the potential for further preclinical development of **2** was severely limited.

**SCHEME 1:**
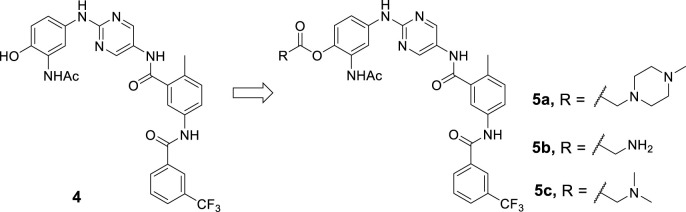
Design strategy for prodrugs **5a-c** based on parent **4**.

Solubility is an essential physicochemical property of therapeutic candidates for drug discovery and development. Poor solubility has a negative impact on oral absorption and can obfuscate compound activity in bioassays in a variety of ways (Kerns and Di, 2004; Sou and Bergström, 2018). To the best of our knowledge, prodrug development has provided a versatile approach to improve the clinical utility of many drugs by improving the physical, biopharmaceutical, or pharmacokinetic properties of the therapeutic agents, including chemical or enzymatic stability, solubility, cell permeability, toxicity, bioavailability, or blood-brain barrier penetration (Silverman and Hayhurst, 2004; Liederer and Borchardt, 2006; Rautio et al., 2008; Vig et al., 2013; Abet et al., 2016). As a result, we decided to investigate a prodrug strategy to circumvent the likely development problem of the BTK inhibitor **2**. Alcohol ester prodrugs have been successfully used in commercial drugs for aqueous solubility enhancement (Hu et al., 2005). Thus, compound **4** with the phenol moiety was identified as the parent molecule derived from lead compound **2**. In this study, we proposed and investigated a rational prodrug approach to introduce a series of soluble groups to the front phenyl ring of parent **4** *via* a metabolically labile ester linkage to improve its physicochemical and pharmacological properties (Scheme 1).

## 2 Results and discussion

### 2.1 Docking studies

**FIGURE 3:**
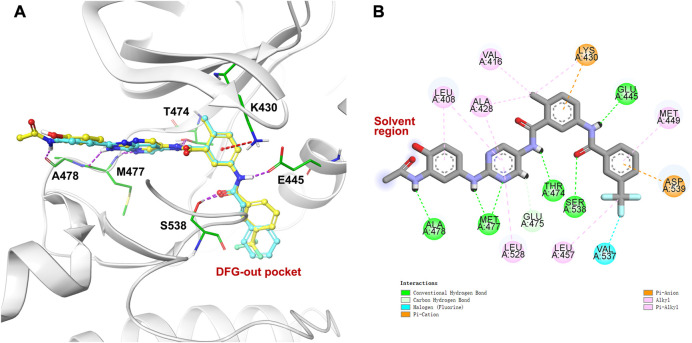
Molecular docking against kinase domain of BTK: **(A)** The binding mode of compound **4** (carbon in yellow) in the DFG-out conformation of BTK (PDB ID: 3pj3), and superimposition with the co-crystallized ligand **1** (carbon in cyan). Hydrogen bonds are visualized as dashed purple lines, π-cation interactions are visulalized as dashed red lines. **(B)** 2D diagram of interactions between compound **4** and BTK.

Compound **4** was first minimized using Ligprep and docked to the BTK kinase domain in the DFG-out conformation (PDB ID: 3pj3) in XP (extra precision) mode using Glide molecular docking software (Schrödinger, 2018) (Figure 3A). It was observed that compound **4** was perfectly superimposed on the original ligand **1**. The spatial interactions show that, as with **1**, several significant hydrogen bonds were formed between the aminopyrimidine moiety and the hinge region. Furthermore, the *N*-acetyl group of the front phenyl ring made an additional hydrogen bond with A478. As for the type-II moiety, the amide created two critical hydrogen bonds with S538 and E445. And the middle phenyl ring formed a π-cation interaction with K430. The hydroxyl group of the front phenyl ring was exposed to the solvent region (Figure 3B), which was identified as a suitable position for prodrug derivatization.

To our knowledge, the most commonly used prodrugs are esters, and it is estimated that enzymatic hydrolysis activates nearly 49% of all marketed prodrugs (Ettmayer et al., 2004). It is usually straightforward for the formation of an ester bond. Once in the body, ubiquitous esterases in the blood, liver, and other organs and tissues readily hydrolyze the ester bond back to the parent drug (Liederer and Borchardt, 2006). Furthermore, the recruitment of the ester moiety may benefit the passive membrane permeability of the prodrug (Taylor, 1996; Beaumont et al., 2003). Therefore, an ester linker was chosen to introduce various polarized tails that have the potential to improve the aqueous solubility of parent **4**, allowing for a more favorable oral or parenteral administration (Scheme 1).

### 2.2 Chemical synthesis

**SCHEME 2:**
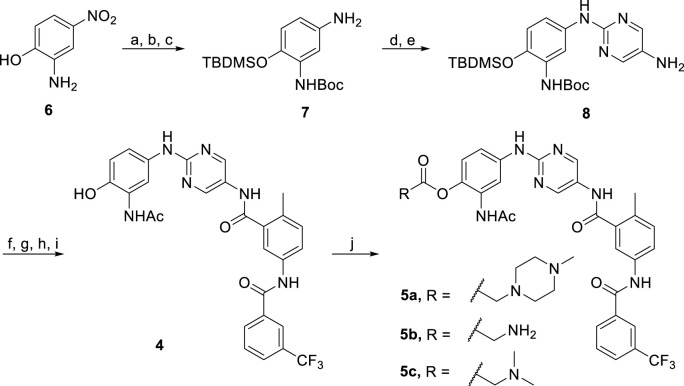
Reagents and conditions: (a) (Boc)_2_O, EtOH, rt, 12 h, 63%; (b) Pd/C, H_2_, MeOH, rt, 12 h, 90%; (c) TBDMSCl, DIEA, CH_2_Cl_2_, rt, 3 h, 67%; (d) 2-Chloro-5-nitropyrimidine, K_2_CO_3_, CH_3_CN, rt, 6 h, 87%; (e) Pd/C, H_2_, MeOH, rt, 12 h, 57%; (f) 2-Methyl-5-(3-(trifluoromethyl)benzamido)benzoic acid, HATU, DIEA, DMF, rt, 10 h, 53%; (g) TFA, CH_2_Cl_2_, rt, 5 h, 70%; (h) Acetyl chloride, DIEA, THF, H_2_O, 0°C to rt, 85%; (i) TBAF, THF, rt, 2 h, 99%; (j) R-COOH, EDCI, DMAP, DMF, 50°C, 10 h, 48–61%.

The synthesis of prodrugs **5a-c** started with the dual protection of substrate **6** with Boc and TBDMS groups, and then the nitro was reduced to the amine group to give compound **7** (Scheme 2). Compound **8** was prepared through the subsequent S_N_2 reaction between **7** and 2-chloro-5-nitropyrimidine, followed by reduction. Coupling of **8** with 2-methyl-5-(3-(trifluoromethyl)benzamido)benzoic acid, followed by the Boc group deprotection, acylation and the subsequent TBDMS group deprotection process gave **4**. Final products **5a-c** were obtained by treating **4** with different carboxylic acids containing piperizine, glycine, or *N,N*-dimethylglycine units in the presence of EDCI and DMAP in DMF. NMR spectroscopy and high resolution mass spectrometry were performed to properly characterize all the final products.

### 2.3 Physicochemical characterization

**SCHEME 3:**
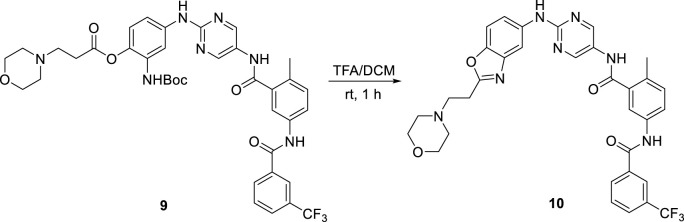
Byproduct **10** yielding process.

It is generally known that the incorporation of piperizine and similar heterocycles, as well as amino acid residues, improves aqueous solubility (Bollini et al., 2013; Walker, 2013). The groups are expected to be protonated at physiological pH, which promotes hydration, and their saturated, non-planar nature allows for loose packing (Vig et al., 2013). Therefore, we focused on amino-containing prodrugs. However, due to the instability of the prodrug, our first efforts to investigate these derivatives were futile. Some derivatives were easily reverted to the parent, even in organic medium, and others were further transformed to other byproducts. For example, deprotection of the Boc group of intermediate **9** under acidic conditions resulted in the unexpected byproduct **10** (Scheme 3). Fortunately, prodrugs **5a-c** were found to be stable in DMSO or aqueous buffer for up to 24 h without significant degradation (Table 1).

**TABLE 1:** Chemical stabilities and aqueous solubilities.

Compd	DMSO^a^ (% remaining)	Buffer, pH 7.4^a^ (% remaining)	S [μM], pH 6.5^b^
**2**	100	NT	2.0
**4**	100	100	6.6
**5a**	100	99	88
**5b**	100	98	22
**5c**	100	NT	4.6

^a^ HPLC, analysis of *in vitro* stability after 24 h at room temperature.

^b^ Thermodynamic solubilities in Britton-Robinson buffer (pH 6.5). Values are the average of two measurements.

The thermodynamic solubilities were determined by the method described in the experimental section. The solubility of compound **2** was very low, only 2.0 μM, as shown in Table 1, which was in agreement with our previous investigations (Li et al., 2014). The solubility of parent **4** improved slightly as compared to **2**. To our delight, prodrug **5a** with a piperizine substitution increased the solubility to 88 μM, while prodrug **5b** with a glycine-containing substituent increased the solubility by thrice. However, when compared to parent **4**, prodrug **5c** containing an *N,N*-dimethylglycine moiety decreased the solubility from 6.6 μM to 4.6 μM.

### 2.4 *In vitro* assessment of prodrugs

**FIGURE 4:**
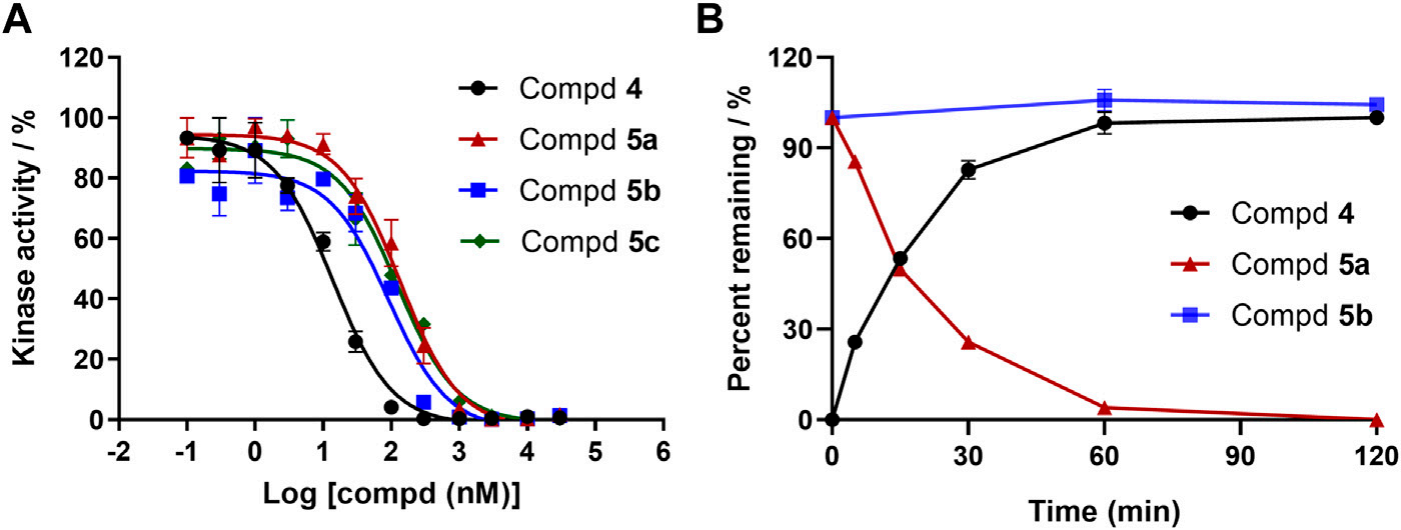
**(A)** Enzymatic activities of parent **4** and prodrugs **5a-c** for BTK. Kinase enzymology assays were carried out in accordance with the protocols described in the HTRF KinEase assays. **(B)** Time-dependent plasma stability assay.

In a cell-free kinase assay, we examined the inhibitory potential of the three prodrugs **5a-c** as well as the parent compound **4** (Figure 4A). Compound **2** was used as a control and yielded IC_50_ = 6 nM, which is in agreement with the literature results (Li et al., 2014). Parent compound **4** was quite potent, as shown in Figure 4A, with an IC_50_ value of 14 nM. Prodrugs **5a-c** had a 10-fold (IC_50_ = 135 nM), 7-fold (IC_50_ = 92 nM), and 9-fold (IC_50_ = 124 nM) lower inhibitory potential for BTK, respectively, compared to parent **4**. These findings are consistent with the docking investigations and point to promising prodrug properties.

To our knowledge, a variety of specialized and non-specific esterases present in the plasma, liver, stomach, and intestine can hydrolyze amino ester bonds (Liederer and Borchardt, 2006). We sought to identify prodrugs with excellent chemical stability and rapid, quantitative conversion to the parent compound to optimize drug exposure while minimizing unproductive metabolism. As a result, prodrugs **5a** and **5b** with enhanced aqueous solubilities were tested to determine their conversion rate to the parent compound in human plasma (Figure 4B). Prodrug **5a** was readily converted to parent **4** in less than 2 h. However, after 2 h of exposure to human plasma, the prodrug **5b** was very stable to ester cleavage, implying that metabolic conversion in the liver is conceivable.

### 2.5 Molecular dynamic studies

The docked pose of **4** at the ATP-binding site in BTK was evaluated for MD simulation studies in order to discover the amino acid residues important for imparting stability to parent **4**, which was identified as active for BTK inhibition in vitro studies. The Schrödinger Desmond tool was used for all MD experiments. The simulation was performed under an isothermal-isobaric (NPT) ensemble for 100 ns.

**FIGURE 5:**
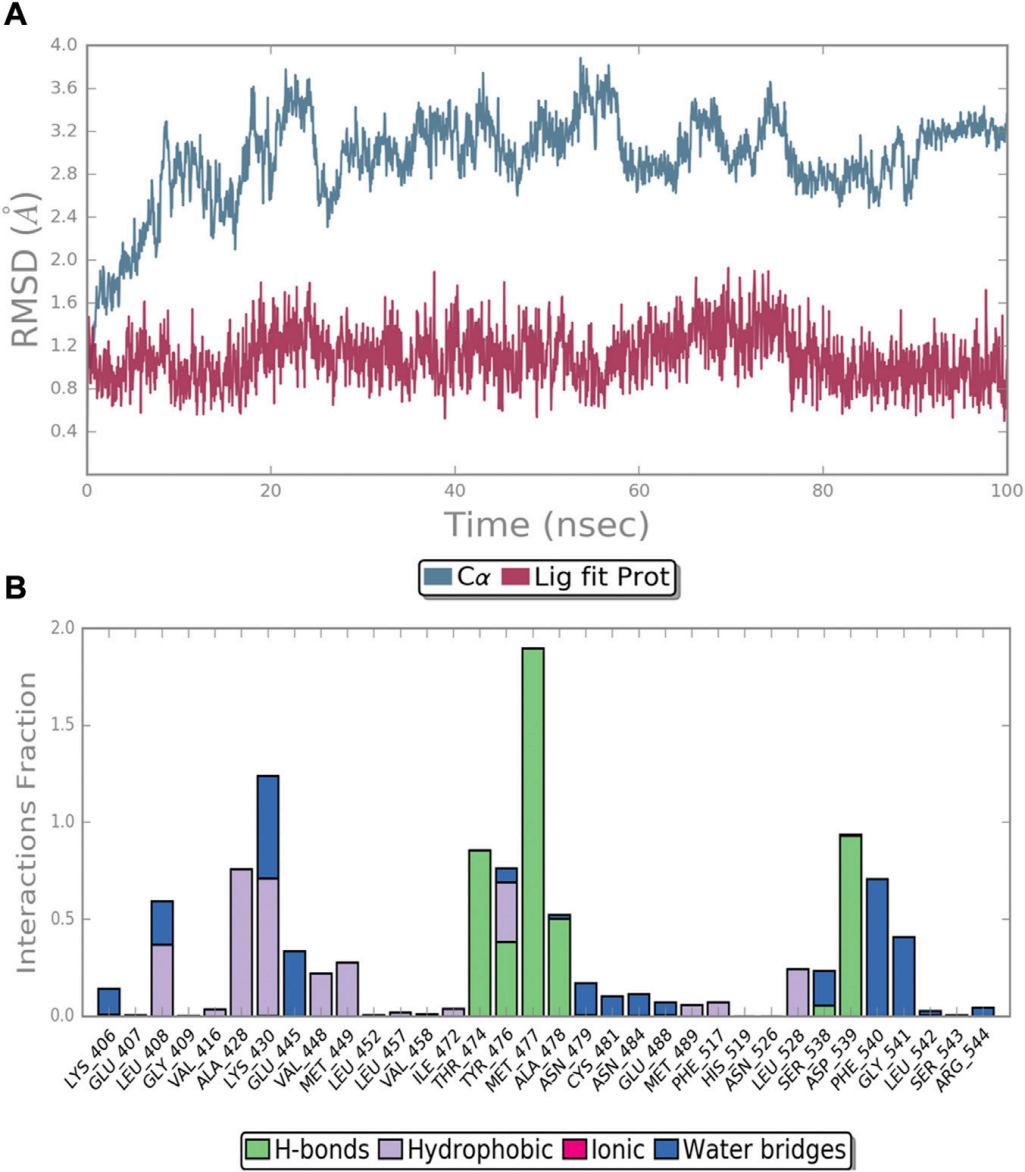
MD simulation of the BTK protein in complex with compound **4**: **(A)** RMSD of protein and ligand; **(B)** protein−ligand contacts during the simulation.

The complex reached stability after 30 ns of simulation run as shown by the RMSD plot (Figure 5A). The MD simulation investigation of compound **4** showed that it formed a stable combination with the protein. The analysis of protein-ligand interactions ranged from 30 ns to 100 ns (Figure 5B). It was found that several critical hydrogen bonds were formed between M477 and the aminopyrimidine moiety. There is an 85% H-bond interaction between T474 and the NH group of the amide moiety attached to the pyrimidine and a 50% H-bond interaction between A478 and the NH group of the acetylamide of the front phenyl ring. Turning to the DFG-out site, the H-bond interaction of D539 remains for 93% of the time. The final binding mode of compound **4** with the kinase domain of BTK after 100 ns dynamic simulation can be found in the supplementary material (Supplementary Figure S1). In addition to the direct hydrogen bonds mentioned above, water-mediated hydrogen bonding also contributed significantly to the stability of the complex. For example, the carbonyl of the acetyl group on the anterior phenyl ring formed two water-mediated hydrogen bonds with N479 and C481, respectively. The amino acid residues G541 and F540 also formed water-mediated hydrogen bonds with the amide moiety attached to the pyrimidine. Similar to the first docking result (Figure 3), a π-cation interaction was reserved between K430 and the middle phenyl ring. Otherwise, alkyl-π interactions between the ligand and L408, A428, and L528 persist during the dynamic process. And the CF_3_-substituted phenyl group extended into the hydrophobic pocket formed by the movement of the DFG sequence. All of the above interactions contribute to the stability of the complex to varying degrees.

## 3 Conclusion

A series of amino ester prodrugs of a BTK inhibitor were designed, synthesized, and evaluated. The inclusion of various solubilizing groups *via* an ester linkage considerably improved the aqueous solubility profile of parent **4** with an insoluble 2,5-diaminopyrimidine scaffold. A cell-free kinase assay indicated that **4** was also a potential BTK inhibitor, but prodrugs **5a-c** had reduced inhibitory potency, indicating promising prodrug properties. *In vitro* evaluation of the conversion of several prodrugs to parent **4** in human plasma led to the identification of **5a** as a potential lead prodrug. Molecular dynamics studies provided evidence to the potent enzymatic inhibitory activity of **4**. The pharmacokinetics, pharmacodynamics, and antitumor effects of these prodrugs will be further investigated in the future experiments.

## 4 Experimental section

### 4.1 Molecular docking

The Schrödinger Protein Preparation Wizard was used to process the protein-ligand structure (PDB ID: 3pj3). The structure of the protein was checked and adjusted, and Prime was used to add the missing residues and the loop regions in the active site. To capture the entire active site environment, a 3D box was built around the initial ligand. The receptor grid was generated using the OPLS 2005 force field. The centroid of the co-crystallized ligand was chosen as the grid center. The LigPrep program from Schrödinger has been used to generate different conformations of the ligands. Molecular docking experiments were carried out utilizing the Schrödinger’s Glide docking module. The co-crystallized ligand (compound **1**) was docked back into BTK’s ATP-binding site (PDB ID: 3pj3) to validate the docking protocol. The docked conformation of compound **1** had similar interactions and binding pose to its initial bound conformation at the binding site. Subsequently, Glide XP (extra precision) mode was used to dock the prepared ligands into the created receptor grids. Glide docking scores and molecular recognition interactions were used to analyze the data. Schrödinger Suite 2018 was used to create the 3D images.

### 4.2 Chemistry

All chemical reagents and solvents were purchased from commercial sources, and used without further purification unless stated. Precoated silica gel 60 GF254 plates were used for analytical thin layer chromatography (TLC). Silica gel (particle size: 0.050–0.075 mm) was used for flash column chromatography. TLC was used to monitor the reactions by the use of UV light as a visualization agent or ethanolic solution of ninhydrin or phosphomolybdic acid as a developer. NMR (nuclear magnetic resonance) spectra data were collected at room temperature using a Bruker Advance-400 (^1^H, 400 MHz; ^13^C, 101 MHz) or Bruker Advance-600 (^1^H, 600 MHz; ^13^C, 151 MHz) spectrometer. The shifts are given in ppm and the coupling constants in Hz. ^1^H NMR data are recorded as: chemical shift (δ, ppm), multiplicity (s, singlet; d, doublet; t, triplet; q, quartet; m, multiplet; br, broad), coupling constant (Hz), and integration. ^13^C NMR data are recorded as chemical shifts (δ, ppm). Mass spectrometry data were acquired with a Bruker Apex IV RTMS. Characterization data of key intermediates and all final compounds, as well as the synthetic route, are provided in the Supplementary Material S1.

### 4.3 Solubility determination assay

Prior to mixing with the appropriate medium, the test chemical was accurately weighed into an appropriately sized screw-capped glass vial. The generated slurry was sonicated at room temperature for 20 min and at 50°C for 15 min, and then agitated at room temperature for at least two days before the crystallinity of the remaining solid was examined using a polarized light microscope. If crystallinity was not observed, more time for equilibration was allowed. The slurry was aliquoted into 1.5 mL centrifuge tubes on the day of analysis and centrifuged at 15,000 rpm for 20 min at 25°C. The supernatant’s concentration was determined by UV-vis (YOKE, T-U7S).

### 4.4 Kinase enzymology assay

The kinase (BTK) was acquired from Carna Biosciences. Kinase acitivity assays were carried out based on Cisbio Bioassays’ HTRF^®^ technology. At room temperature, kinase and substrate were combined with compounds in varied concentrations, followed by adding ATP to activate the enzymatic reactions. EDTA solution was added to stop the reactions after 1 h, followed by the addition of streptavidin XL665 conjugates and anti-phosphotyrosine antibodies. The mixtures were incubated for an additional 1 h at room temperature before reading on a plate reader (EnVision^®^, Perkin Elmer). GraphPad Prism was used to analyze the data.

### 4.5 Plasma stability assay

The *in vitro* stability in human plasma for the test compounds was investigated using procaine as the reference compound. The frozen human plasma was quickly thawed at 37°C. The spiking solution was made by combining 10 µL of a 10 mM compound stock solution with 990 µL of DMSO. The plasma and spiking solution were pre-warmed at 37°C for 5 min. Then 7 µL of the pre-warmed spiking solution was added into the 693 µL plasma evenly. The tests were carried out in duplicate in a 37°C shaking water bath. At 0, 5, 15, 30, 60, and 120 min, 100 µL samples were collected and added to a 400 µL solution containing an internal standard (IS). After quenching, the samples were vortexed for 5 min (600 rpm) and then centrifuged at 4,000 rpm for 20 min. LC-MS was used to analyze the clear supernatants. The values are the mean of two separate experiments.

### 4.6 Molecular dynamic studies

Molecular dynamics simulations using the Desmond package (Schrödinger, 2018) have been carried out for the study of the conformational changes of the ligand-protein complex in the solvent environment. The docked complex simulation was run using OPLS force field parameters. The protein structure was prepared by the use of Protein Preparation Wizard in Schrödinger, and solvated in an orthorhombic box with TIP3P water molecules at least 10 Å spacing. The MD simulation was performed under an isothermal isobaric ensemble (NPT) with a pressure of 1 atm, a temperature of 300 K, and a relaxation time of the thermostat of 200 ps. The simulation was run for 100 ns with the trajectory recorded every 50 ps. The Simulation Interactions Diagram (SID) was used to analyze the MD trajectories.

## Data Availability

The original contributions presented in the study are included in the article/Supplementary Material, further inquiries can be directed to the corresponding author.
