# Supplementary material for: Design, synthesis and evaluation of a series of potential prodrugs of a Bruton’s tyrosine kinase (BTK) inhibitor
Source: Front Pharmacol. 2023 Mar 8;14:1162216. doi: 10.3389/fphar.2023.1162216 (PMC10031131; doi:10.3389/fphar.2023.1162216)
Supplement: Supplementary file 1 [file DataSheet1.docx]

Supplementary Material

**Design, synthesis and evaluation of a series of potential prodrugs of a Bruton’s tyrosine kinase (BTK) inhibitor**

Zhou-Peng Xiao^1^, Min Liao^1^, Xue-Juan Huang^1^, Yu-Tong wang^1^, Xiao-Cui Lan^1^, Xue-Ying Wang^2^ and Xi-Tao Li^1^*

^1^School of Pharmaceutical Sciences (Shenzhen), Sun Yat-sen University, Shenzhen 518107, China

^2^BayRay Innovative Center, Shenzhen Bay Laboratory, Shenzhen 518032, China

*** Correspondence:**

Corresponding Author

lixt78@mail.sysu.edu.cn

1. **Chemistry**
   1. **General information**

All chemical reagents and solvents were purchased from commercial sources, and used without further purification unless stated. Precoated silica gel 60 GF254 plates were used for analytical thin layer chromatography (TLC). Silica gel (particle size: 0.050-0.075 mm) was used for flash column chromatography. TLC was used to monitor the reactions by the use of UV light as a visualization agent or ethanolic solution of ninhydrin or phosphomolybdic acid as a developer. NMR (nuclear magnetic resonance) spectra data were collected at room temperature using a Bruker Advance-400 (^1^H, 400 MHz; ^13^C, 101 MHz) or Bruker Advance-600 (^1^H, 600 MHz; ^13^C, 151 MHz) spectrometer. The shifts are given in ppm and the coupling constants in Hz. ^1^H NMR data are recorded as: chemical shift (δ, ppm), multiplicity (s, singlet; d, doublet; t, triplet; q, quartet; m, multiplet; br, broad), coupling constant (Hz), and integration. ^13^C NMR data are recorded as chemical shifts (δ, ppm). Mass spectrometry data were acquired with a Bruker Apex IV RTMS. Characterization data of key intermediates and all final compounds, as well as the synthetic route, are provided in the supplementary material.

- 1. **General synthetic route for 5a-c：**

- 1. **Example:** Synthesis of **5a:**

*Step 1*. Synthesis of **S3**: To a solution of **S2** (3.50 g, 16.8 mmol) in mixed solvent of 80 mL water and 20 mL tetrahydrofuran was added NaOH (0.672 g, 16.8 mol) and the mixture was cooled down to 0 °C. Then **S1** (2.54 g, 16.8 mmol) was added dropwise with stirring. The mixture was warmed to room temperature and stirred for 6 hours. Then the reaction was quenched by adding water (20 mL), and the resulting organic phase was subsequently extracted three times with ethyl acetate. The combined extracts were washed with brine, and dried over anhydrous Na_2_SO_4_, concentrated and purified over silica gel flash column chromatography (eluent: petroleum ether/ethyl acetate = 3:1 – 1:1) to afford the product (3.80 g, 70%). **^1^H NMR** (400 MHz, DMSO-*d*_6_) δ 10.53 (s, 1H), 8.36 – 8.25 (m, 2H), 8.25 (d, *J* = 2.4 Hz, 1H), 7.96 (d, *J* = 7.8 Hz, 1H), 7.90 (dd, *J* = 8.3, 2.4 Hz, 1H), 7.78 (t, *J* = 7.8 Hz, 1H), 7.29 (d, *J* = 8.3 Hz, 1H), 2.49 (s, 3H).

*Step 2*. Synthesis of **S4**: To a solution of **6** (2.00 g, 13.0 mmol) in 40 mL EtOH was added (Boc)_2_O (5.60 g, 26.0 mol). The mixture was stirred at room temperature for 12 h. Then the reaction was quenched by adding water (10 mL), and the resulting organic phase was subsequently extracted three times with ethyl acetate. The combined extracts were washed with brine, and dried over anhydrous Na_2_SO_4_, concentrated and purified over silica gel flash column chromatography (eluent: petroleum ether/ethyl acetate = 4:1 – 2:1) to afford the product (2.10 g, 63%).

*Step 3*. Synthesis of **S5**: To a solution of **S4** (0.500 g, 1.97 mmol) in 10 mL MeOH was added 10% (w/w) Pd/C (0.250 g). The reaction mixture was stirred under hydrogen atmosphere (balloon) at room temperature for 12 h, and then filtered through Celite^®^ washing with MeOH. Evaporation of the filtrate gave a residue, which was purified over silica gel flash column chromatography (CH_2_Cl_2_/MeOH = 100:1 – 20:1) to afford the product (0.400 g, 90%).

*Step 4*. Synthesis of **7**: To a solution of **S5** (3.20 g, 13.7 mmol) in 80 mL DCM was added DIEA (4.53 mL, 27.3 mmol) and TBDMSCl (3.10 g, 20.5 mmol). The reaction was stirred at room temperature for 3 h, and quenched by the addition of H_2_O (10 mL). The mixture was diluted with EtOAc, washed with H_2_O and brine, dried over anhydrous Na_2_SO_4_ and concentrated under reduced pressure. Purification over silica gel flash column chromatography (eluent: petroleum ether/ethyl acetate = 6:1 – 4:1) to afford the product (3.20 g, 67%).

*Step 5*. Synthesis of **S6**: To a solution of **7** (3.20 g, 9.18 mmol) in 50 mL CH_3_CN was added 2-chloro-5-nitropyrimidine (1.45 g, 9.18 mmol) and K_2_CO_3_ (3.81 g, 27.5 mmol). The mixture was stirred at room temperature for 6 h. Then the reaction was quenched by adding water (5 mL), and the resulting organic phase was subsequently extracted three times with ethyl acetate. The combined extracts were washed with brine, and dried over anhydrous Na_2_SO_4_, concentrated and purified over silica gel flash column chromatography (eluent: petroleum ether/ethyl acetate = 12:1 – 6:1) to afford the product (3.70 g, 87%).

*Step 6*. Synthesis of **8**: To a solution of **S6** (4.00 g, 8.67 mmol) in 50 mL MeOH was added 10% (w/w) Pd/C (4.00 g). The reaction mixture was stirred under hydrogen atmosphere (balloon) at room temperature for 12 h, and then filtered through Celite^®^ washing with MeOH. Evaporation of the filtrate gave a residue, which was purified over silica gel flash column chromatography (eluent: petroleum ether/ethyl acetate = 4:1 – 1:1) to afford the product (2.10 g, 57%). **^1^H NMR** (400 MHz, DMSO-*d*_6_) δ 8.74 (s, 1H), 7.91 (s, 2H), 7.74 (d, *J* = 2.7 Hz, 1H), 7.61 (s, 1H), 7.29 (dd, *J* = 8.8, 2.7 Hz, 1H), 6.71 (d, *J* = 8.7 Hz, 1H), 4.71 (s, 2H), 1.42 (s, 9H), 0.95 (s, 9H), 0.13 (s, 6H).

*Step 7*. Synthesis of **S7**: To a solution of **8** (1.80 g, 4.20 mmol) in 30 mL DMF was added **S3** (1.63 g, 5.04 mmol), HATU (2.40 g, 6.30 mmol) and DIEA (2.1 mL, 12.6 mmol). The reaction mixture was stirred at room temperature for 10 h. Then the reaction was quenched by adding water (5 mL), and the resulting organic phase were subsequently extracted three times with ethyl acetate. The combined extracts were washed with brine, and dried over anhydrous Na_2_SO_4_, concentrated and purified over silica gel flash column chromatography (eluent: petroleum ether/ethyl acetate = 4:1 – 1:2) to afford the product (1.60 g, 53%). **^1^H NMR** (400 MHz, CDCl_3_) δ 8.86 (s, 1H), 8.67 (s, 1H), 8.57 (s, 2H), 8.18 (s, 1H), 8.11 (s, 1H), 8.01 (d, *J* = 7.0 Hz, 1H), 7.92 (d, *J* = 2.3 Hz, 1H), 7.73 (d, *J* = 7.8 Hz, 1H), 7.58 (dd, *J* = 8.3, 2.3 Hz, 1H), 7.52 (t, *J* = 7.8 Hz, 1H), 7.19 (d, *J* = 8.3 Hz, 1H), 7.04 (s, 1H), 6.69 (d, *J* = 8.6 Hz, 1H), 2.42 (s, 3H), 1.46 (s, 9H), 1.03 (s, 9H), 0.22 (s, 6H).

*Step 8*. Synthesis of **S8**: To a solution of **S7** (0.250 g, 0.340 mmol) in 15 mL DCM was added 15 mL TFA. The mixture was stirred at room temperature for 1 hours. Then the reaction was quenched by adding saturated aqueous sodium bicarbonate solution (1 mL), and the resulting organic phase was subsequently extracted three times with ethyl acetate. The combined extracts were washed with brine, and dried over anhydrous Na_2_SO_4_, concentrated and purified over silica gel flash column chromatography (eluent: petroleum ether/ethyl acetate = 5:1 – 1:1) to afford the product (0.150 g, 70%).

*Step 9*. Synthesis of **S9**: To a solution of **S8** (0.330 g, 0.520 mmol) in mixed solvent of 4.0 mL THF and 1.0 mL water was added DIEA (0.130 g, 1.04 mmol), and the mixture was cooled down to 0 °C. The acetyl chloride (0.060 g, 0.780 mmol) was added dropwise with stirring. After the mixture was warmed to room temperature and it was stirred for 0.5 h. Then the reaction was quenched by adding water (1 mL), and the resulting organic phase was subsequently extracted six times with ethyl acetate. The combined extracts were washed with brine, and dried over anhydrous Na_2_SO_4_, concentrated and purified over silica gel flash column chromatography (eluent: petroleum ether/ethyl acetate = 4:1 – 1:1) to afford the product (0.300 g, 85%).

*Step 10*. Synthesis of **4**: To a solution of **S9** (0.050 g, 0.070 mmol) in 1.0 mL THF was added 0.5 mL TBAF (1.0 M in THF). The mixture was stirred at room temperature for 2 h. Then the reaction was quenched by rapidly adding saturated aqueous sodium bicarbonate solution (1 mL), and the resulting organic phase was subsequently extracted three times with ethyl acetate. The combined extracts were washed with brine, and dried over anhydrous Na_2_SO_4_, concentrated and purified over silica gel flash column chromatography (eluent: petroleum ether/ethyl acetate = 2:1 – 1:2) to afford the product (0.040 g, 99%). **^1^H NMR** (400 MHz, DMSO-*d*_6_) δ 10.57 (s, 1H), 10.34 (s, 1H), 9.47 (s, 1H), 9.34 (s, 1H), 9.30 (s, 1H), 8.72 (s, 2H), 8.33 (s, 1H), 8.29 (d, *J* = 7.9 Hz, 1H), 7.99 (d, *J* = 7.8 Hz, 1H), 7.93 (dd, *J* = 7.7, 2.5 Hz, 2H), 7.85 (dd, *J* = 8.3, 2.3 Hz, 1H), 7.81 (t, *J* = 7.8 Hz, 1H), 7.33 (d, J = 8.4 Hz, 1H), 7.28 (dd, *J* = 8.7, 2.6 Hz, 1H), 6.79 (d, *J* = 8.7 Hz, 1H), 2.39 (s, 3H), 2.11 (s, 3H). **^13^C NMR** (101 MHz, DMSO-*d*_6_) δ 167.95, 164.42, 157.48, 150.52, 143.60, 137.09, 136.98, 135.97, 132.83, 132.33, 131.45, 131.33, 130.30, 129.69 (q, *J* = 32.45 Hz), 128.76 (q, *J* = 3.47 Hz), 126.38, 126.11, 124.64 (q, *J* = 4.01 Hz), 124.45 (q, *J* = 273.52 Hz), 122.19, 119.72, 117.07, 116.52, 114.78, 23.02, 19.30. **HRMS** (ESI) m/z calcd. for C_28_H_23_F_3_N_6_O_4_Na [M+Na]^+^ 587.1631, found 587.1622.

*Step 11*. Synthesis of **5a**: To a solution of **4** (0.050 g, 0.090 mmol) in 1.0 mL DMF was added 2-(4-methylpiperazin-1-yl) acetic acid (0.020 g, 0.110 mmol), EDCI (0.030 g 0.140 mmol), DMAP (0.001 g, 0.009 mmol). The mixture was stirred at 50 ℃ for 10 hours. Then the reaction was quenched by adding saturated aqueous sodium bicarbonate solution (1 mL), and the resulting organic phase was subsequently extracted three times with ethyl acetate. The combined extracts were washed with brine, and dried over anhydrous Na_2_SO_4_, concentrated and purified over silica gel flash column chromatography (CH_2_Cl_2_/MeOH = 50:1 – 10:1) to afford the product (0.030 g, 48%). **^1^H NMR** (400 MHz, DMSO-*d*_6_) δ 10.61 (s, 1H), 10.44 (s, 1H), 9.72 (s, 1H), 9.35 (s, 1H), 8.80 (s, 2H), 8.53 (d, *J* = 2.6 Hz, 1H), 8.34 (s, 1H), 8.30 (d, *J* = 8.0 Hz, 1H), 7.99 (d, *J* = 7.8 Hz, 1H), 7.94 (d, *J* = 2.3 Hz, 1H), 7.86 (dd, *J* = 8.3, 2.3 Hz, 1H), 7.81 (t, *J* = 7.8 Hz, 1H), 7.50 (dd, *J* = 8.9, 2.6 Hz, 1H), 7.34 (d, *J* = 8.4 Hz, 1H), 7.09 (d, *J* = 8.9 Hz, 1H), 3.17 (s, 2H), 2.57 (d, *J* = 27.3 Hz, 7H), 2.39 (s, 3H), 2.36 (s, 3H), 2.30 (s, 3H). **^13^C NMR** (101 MHz, DMSO-*d*_6_) δ 169.37, 168.32, 168.03, 164.43, 156.95, 150.32, 138.97, 137.00, 135.95, 135.07, 132.34, 131.46, 131.35, 130.30, 130.11, 129.68 (q, *J* = 32.32 Hz), 128.77 (q, *J* = 3.83 Hz), 126.93, 124.66 (q, *J* = 3.94 Hz), 124.45 (q, *J* = 273.52), 123.10, 122.83, 122.27, 119.75, 114.91, 112.31, 61.56, 54.91, 52.55, 45.98, 21.61, 19.30. **HRMS** (ESI) m/z calcd. for C_35_H_35_F_3_N_8_O_5_Na [M+Na]^+^ 727.2580, found 727.2567.

**2-Acetamido-4-((5-(2-methyl-5-(3-(trifluoromethyl)benzamido)benzamido)pyrimidin-2-yl)amino)phenyl glycinate (5b)**

**^1^H NMR** (400 MHz, DMSO-*d*_6_) δ 10.58 (s, 1H), 10.34 (s, 1H), 9.33 (s, 1H), 9.18 (s, 1H), 8.72 (s, 2H), 8.33 (s, 2H), 8.29 (d, *J* = 8.1 Hz, 1H), 8.16 (d, *J* = 2.5 Hz, 1H), 7.99 (d, *J* = 7.8 Hz, 1H), 7.92 (d, *J* = 2.3 Hz, 1H), 7.86 (dd, *J* = 8.3, 2.3 Hz, 1H), 7.81 (t, *J* = 7.8 Hz, 1H), 7.33 (d, *J* = 8.4 Hz, 1H), 7.26 (dd, *J* = 8.7, 2.6 Hz, 1H), 6.79 (d, *J* = 8.7 Hz, 1H), 3.91 (d, *J* = 5.9 Hz, 2H), 2.39 (s, 3H), 1.91 (s, 3H). **^13^C NMR** (101 MHz, DMSO-*d*_6_) δ 170.35, 168.47, 167.95, 164.42, 157.50, 150.52, 142.96, 137.09, 136.99, 135.96, 132.78, 132.34, 131.44, 131.33, 130.30, 129.68 (q, *J* = 28.50), 128.75 (q, *J* = 3.62), 126.09, 126.06, 124.65 (q, *J* = 3.78), 124.46 (q, *J* = 273.31), 122.19, 119.73, 116.62, 115.68, 114.12, 46.12, 22.89, 19.30. **HRMS** (ESI) m/z calcd. for C_30_H_26_F_3_N_7_O_5_Na [M+Na]^+^ 621.1845, found 621.1834.

**2-Acetamido-4-((5-(2-methyl-5-(3-(trifluoromethyl)benzamido)benzamido)pyrimidin-2-yl)amino)phenyl dimethylglycinate (5c)**

**^1^H NMR** (400 MHz, DMSO-*d*_6_) δ 10.58 (s, 1H), 10.43 (s, 1H), 9.71 (s, 1H), 9.37 (s, 1H), 8.80 (s, 2H), 8.43 (d, *J* = 2.6 Hz, 1H), 8.33 (s, 1H), 8.29 (d, *J* = 7.9 Hz, 1H), 7.99 (d, *J* = 7.8 Hz, 1H), 7.93 (d, *J* = 2.3 Hz, 1H), 7.86 (dd, *J* = 8.3, 2.3 Hz, 1H), 7.81 (t, *J* = 7.8 Hz, 1H), 7.51 (dd, *J* = 8.8, 2.7 Hz, 1H), 7.34 (d, *J* = 8.3 Hz, 1H), 7.08 (d, *J* = 8.8 Hz, 1H), 3.07 (s, 2H), 2.39 (s, 3H), 2.36 – 2.26 (m, 9H). **^13^C NMR** (101 MHz, DMSO-*d*_6_) δ 169.49, 168.57, 168.01, 164.43, 156.95, 150.32, 139.00, 137.00, 135.96, 135.37, 132.33, 131.47, 131.36, 130.30, 130.11, 129.69 (q, *J* = 31.94), 128.76 (q, *J* = 2.93), 126.92, 124.64 (q, *J* = 3.84), 124.45 (q, *J* = 272.68), 123.09, 122.26, 119.74, 114.96, 112.64, 63.40, 45.83, 20.98, 19.29. **HRMS** (ESI) m/z calcd. for C_32_H_30_F_3_N_7_O_5_Na [M+Na]^+^ 672.2158, found 672.2149.

**2-((*Tert*-butoxycarbonyl)amino)-4-((5-(2-methyl-5-(3-(trifluoromethyl)benzamido)benzamido)pyrimidin-2-yl)amino)phenyl 3-morpholinopropanoate (9)**

**^1^H NMR** (400 MHz, DMSO-*d*_6_) δ 10.57 (s, 1H), 10.41 (s, 1H), 9.64 (s, 1H), 8.78 (s, 2H), 8.54 (s, 1H), 8.32 (s, 1H), 8.28 (d, *J* = 7.9 Hz, 1H), 8.06 (d, *J* = 2.6 Hz, 1H), 7.97 (d, *J* = 7.8 Hz, 1H), 7.92 (d, *J* = 2.3 Hz, 1H), 7.84 (dd, *J* = 8.3, 2.3 Hz, 1H), 7.79 (t, *J* = 7.8 Hz, 1H), 7.47 (dd, *J* = 8.9, 2.6 Hz, 1H), 7.32 (d, *J* = 8.4 Hz, 1H), 6.96 (d, *J* = 8.8 Hz, 1H), 3.58 (t, *J* = 4.6 Hz, 4H), 2.73 (d, *J* = 6.1 Hz, 2H), 2.70 – 2.63 (m, 2H), 2.43 (t, *J* = 4.7 Hz, 4H), 2.38 (s, 3H), 1.46 (s, 9H).

**2-Methyl-*N*-(2-((2-(2-morpholinoethyl)benzo[*d*]oxazol-5-yl)amino)pyrimidin-5-yl)-5-(3-(trifluoromethyl)benzamido)benzamide (10)**

**^1^H NMR** (400 MHz, Methanol-*d*_4_) δ 8.76 (s, 2H), 8.26 (d, *J* = 6.2 Hz, 2H), 8.21 (d, *J* = 7.9 Hz, 1H), 7.97 (s, 1H), 7.90 (s, 1H), 7.73 (t, *J* = 7.8 Hz, 1H), 7.67 (dd, *J* = 8.3, 2.3 Hz, 1H), 7.51 (d, *J* = 3.4 Hz, 2H), 7.33 (d, *J* = 8.3 Hz, 1H), 3.85 (t, *J* = 4.9 Hz, 4H), 3.47 (d, *J* = 6.9 Hz, 2H), 3.39 (d, *J* = 6.8 Hz, 2H), 3.14 (s, 4H), 2.46 (s, 3H). **^13^C NMR** (101 MHz, Methanol-*d*_4_) δ 169.43, 165.71, 157.32, 150.58, 146.33, 140.77, 137.66, 136.14, 135.65, 132.20, 130.96, 130.2995 (q, *J* = 20.15), 130.11, 129.33, 128.07 (q, *J* = 8.5), 124.16 (q, *J* = 4.45), 124.29 (q, *J* = 328.22), 122.66, 119.70, 117.55, 109.85, 109.29, 64.57, 53.89, 52.44, 23.73, 17.89. **HRMS** (ESI) m/z calcd. for C_33_H_31_F_3_N_7_O_4_ [M+H]^+^ 646.2390, found 646.2388.

1. **NMR Spectra**


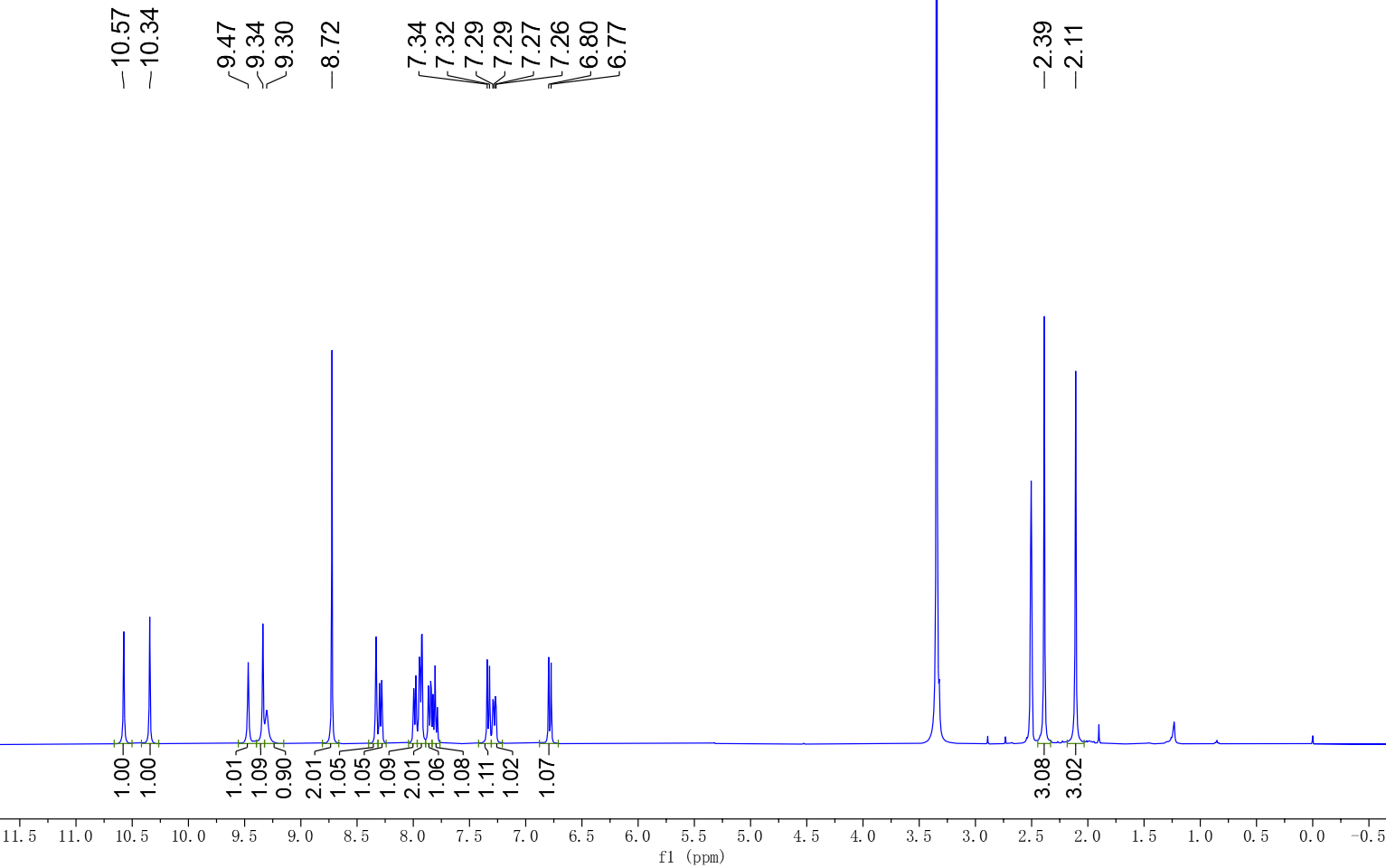


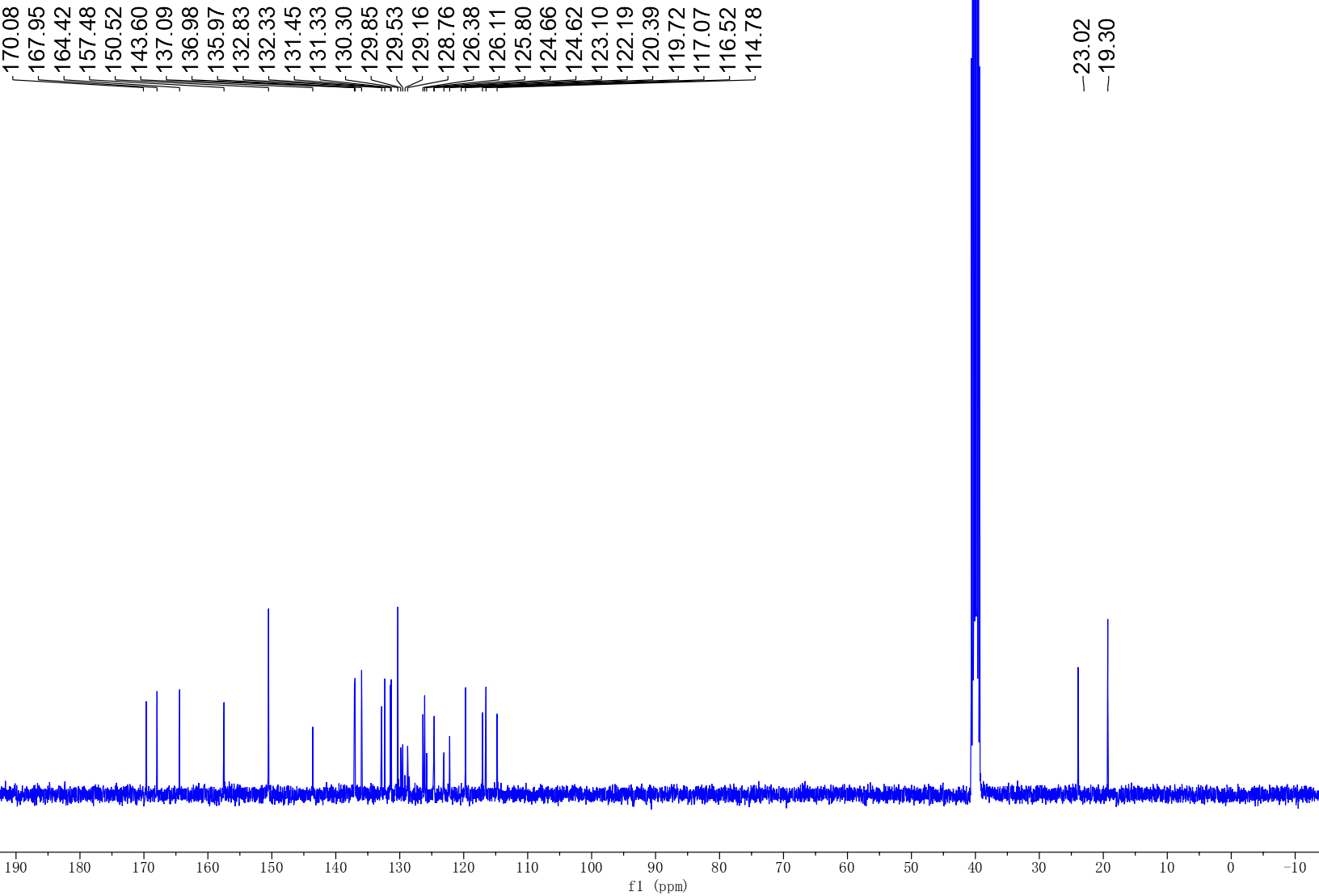


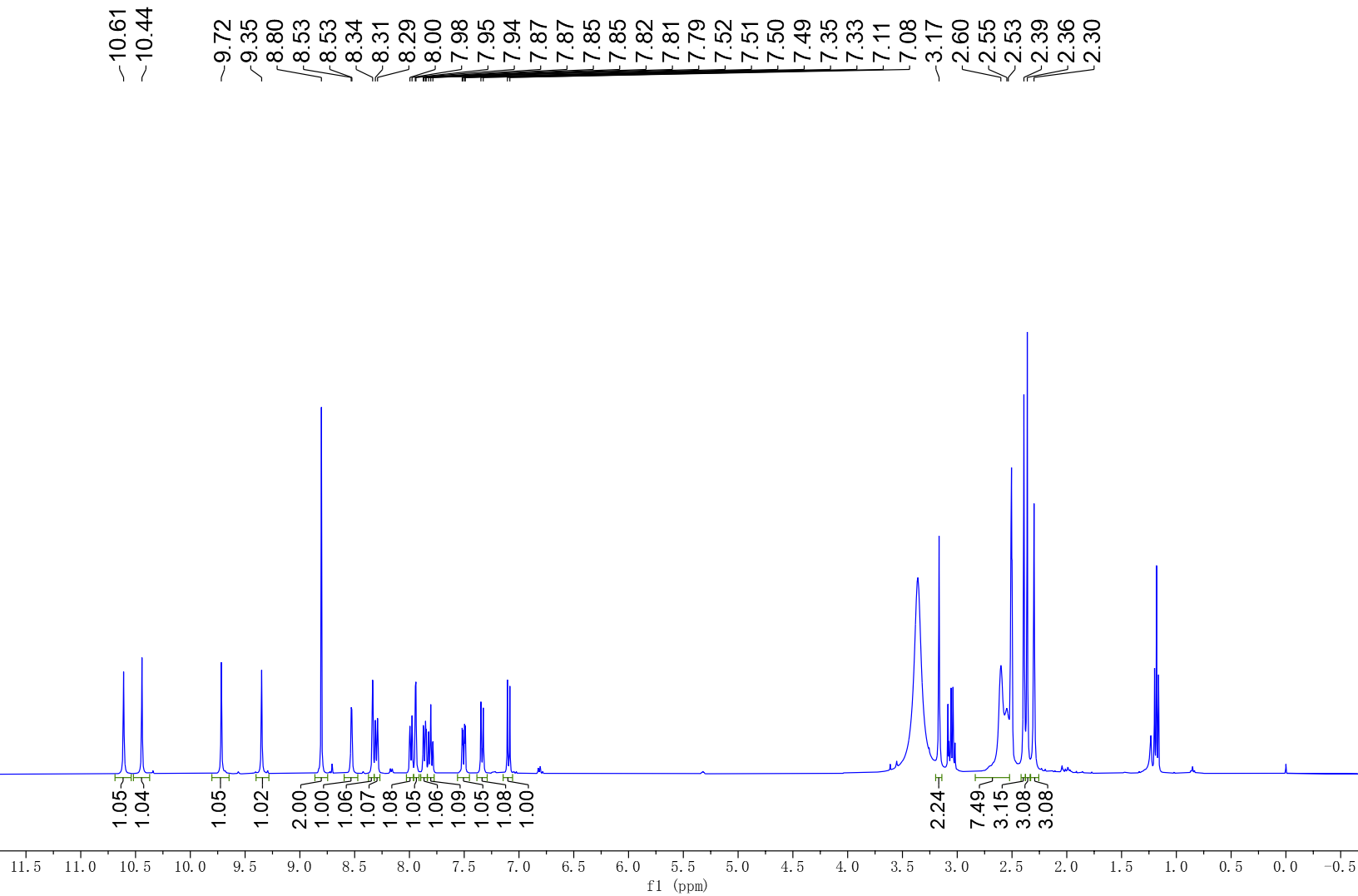


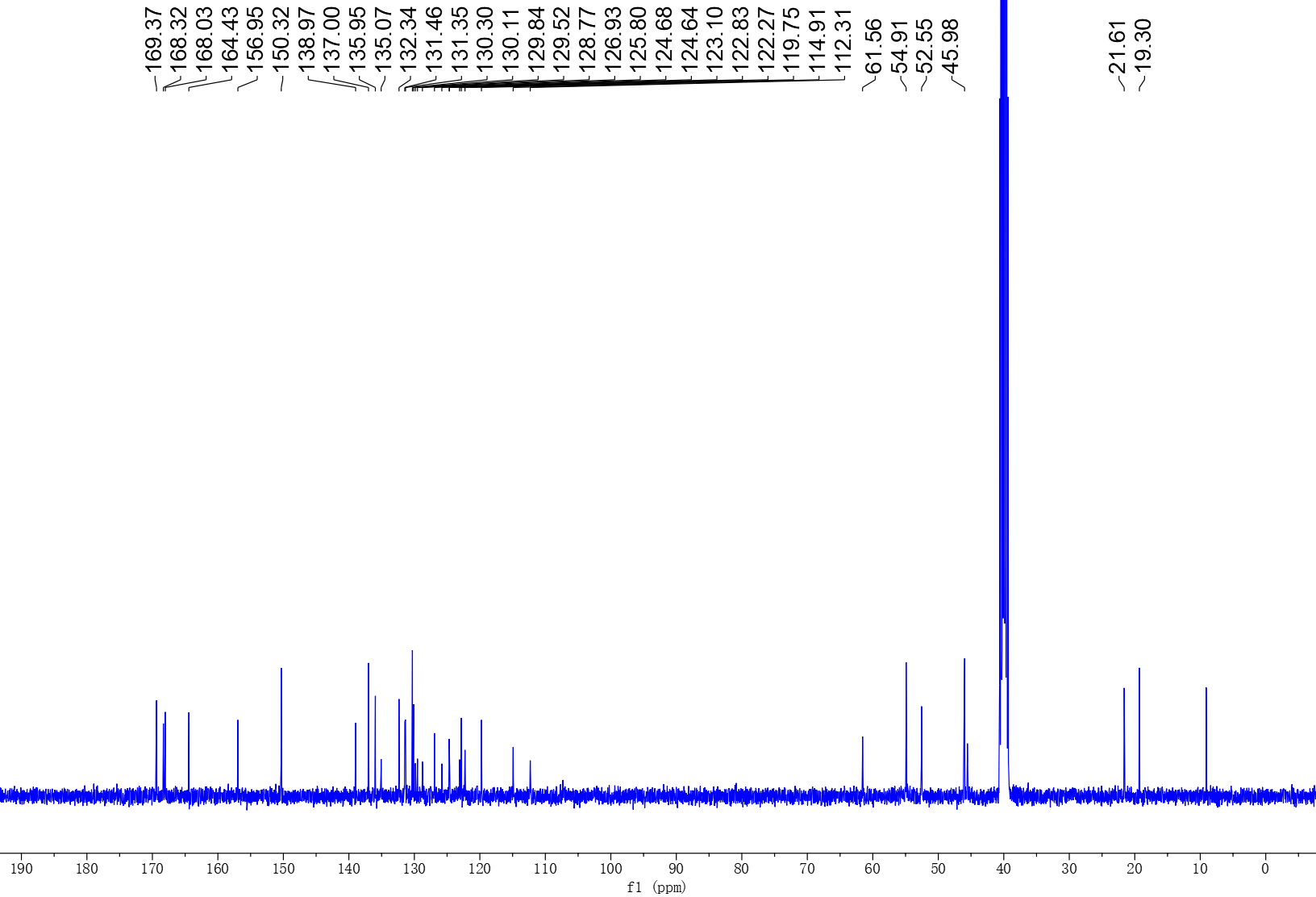


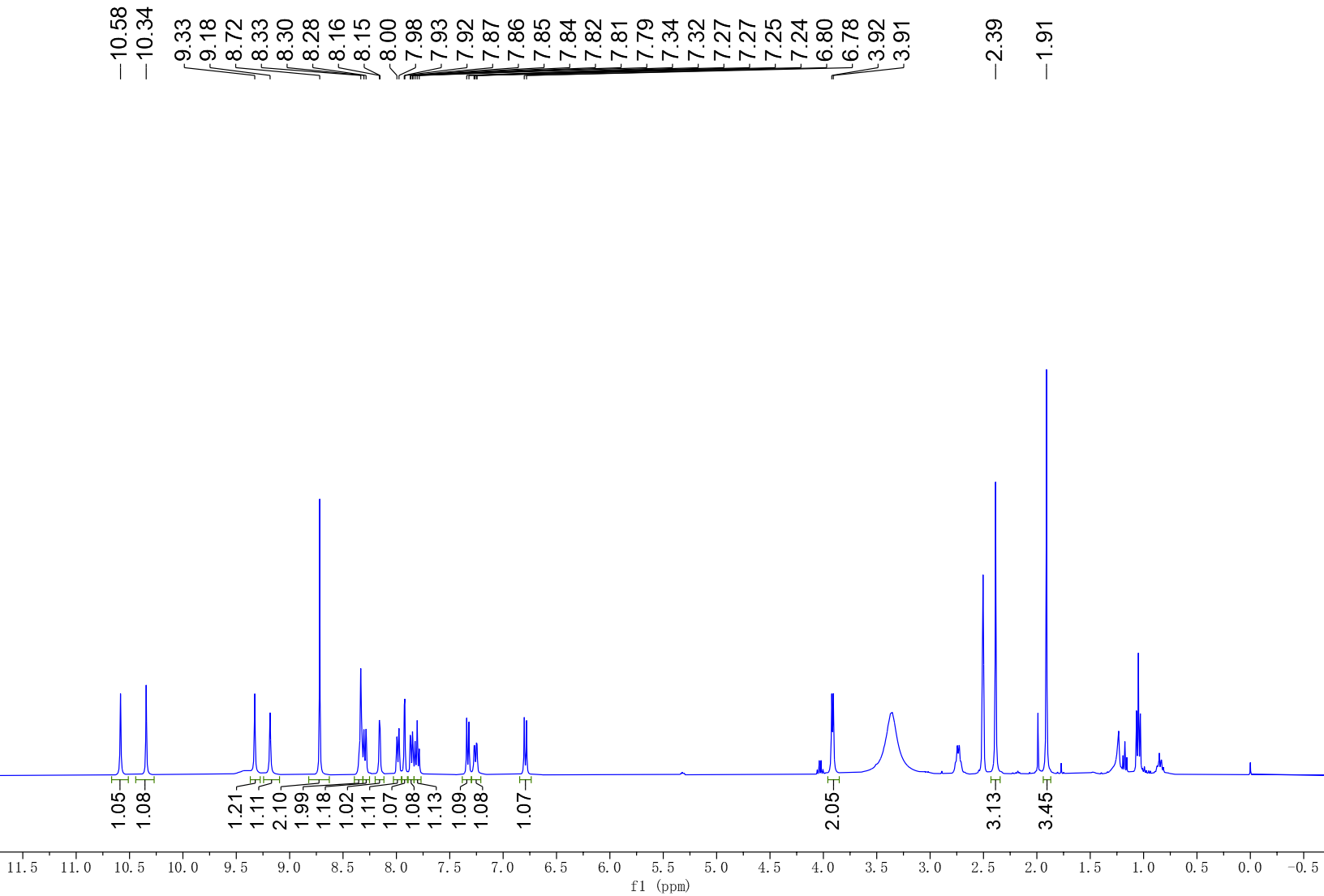


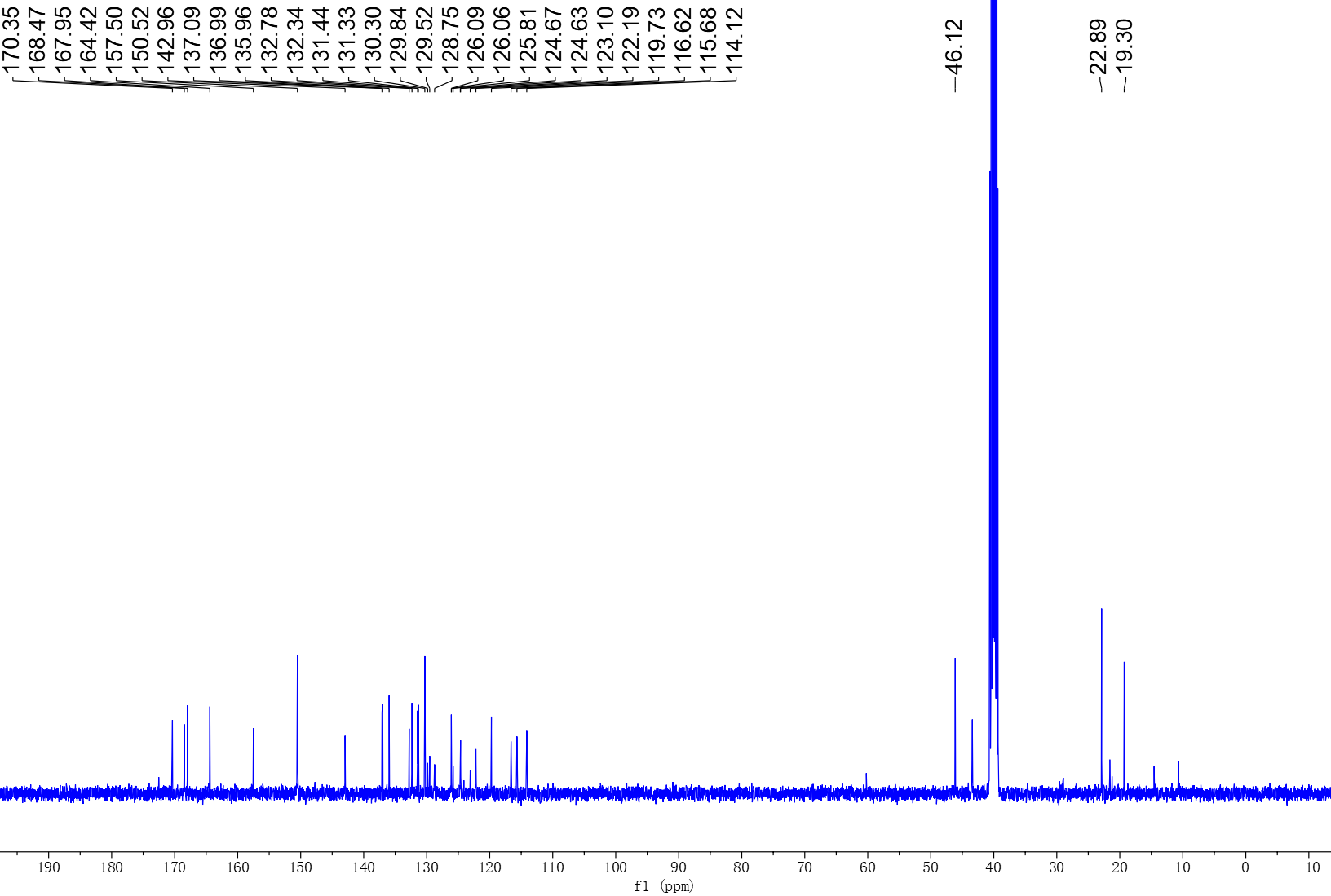


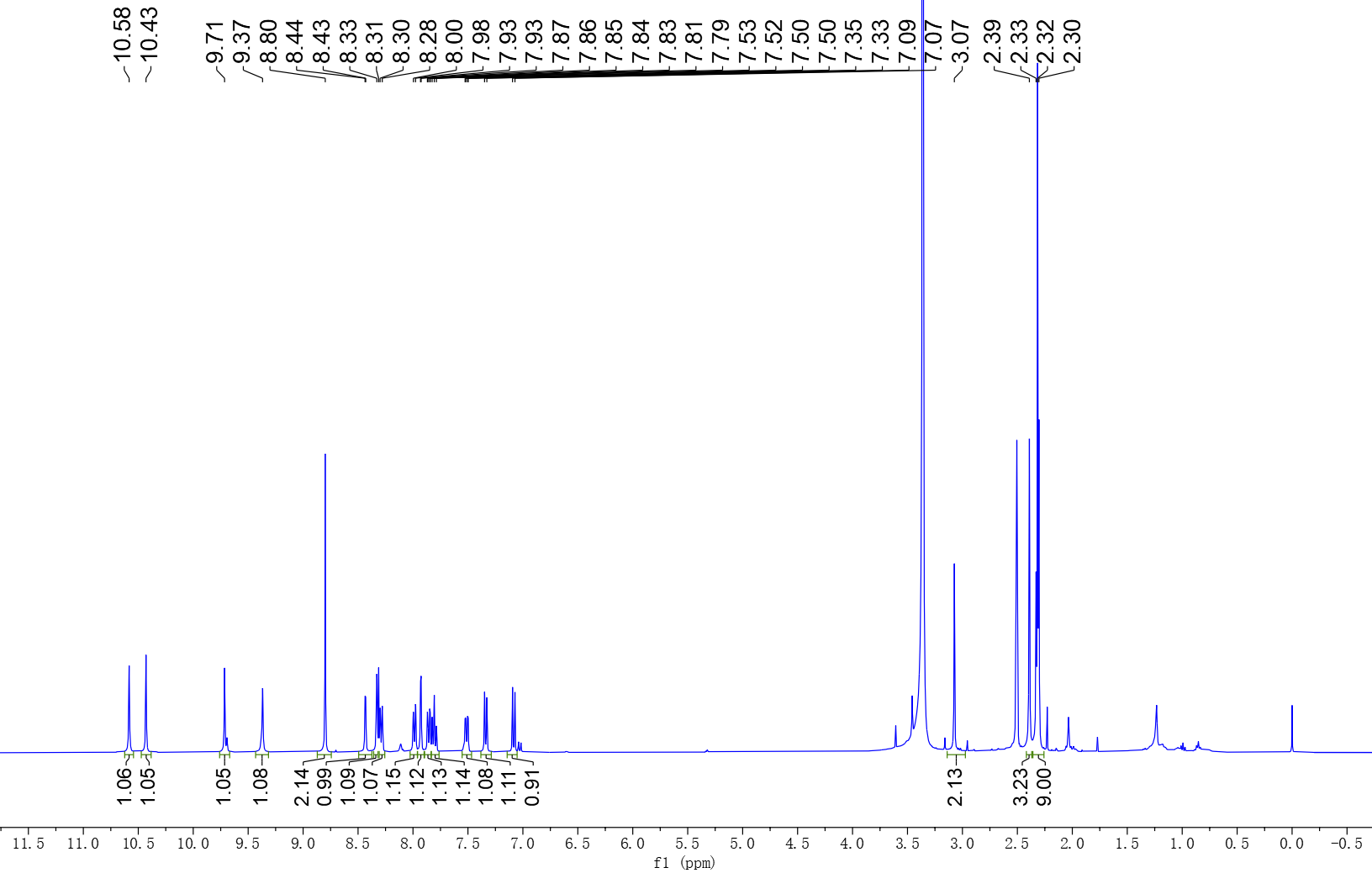


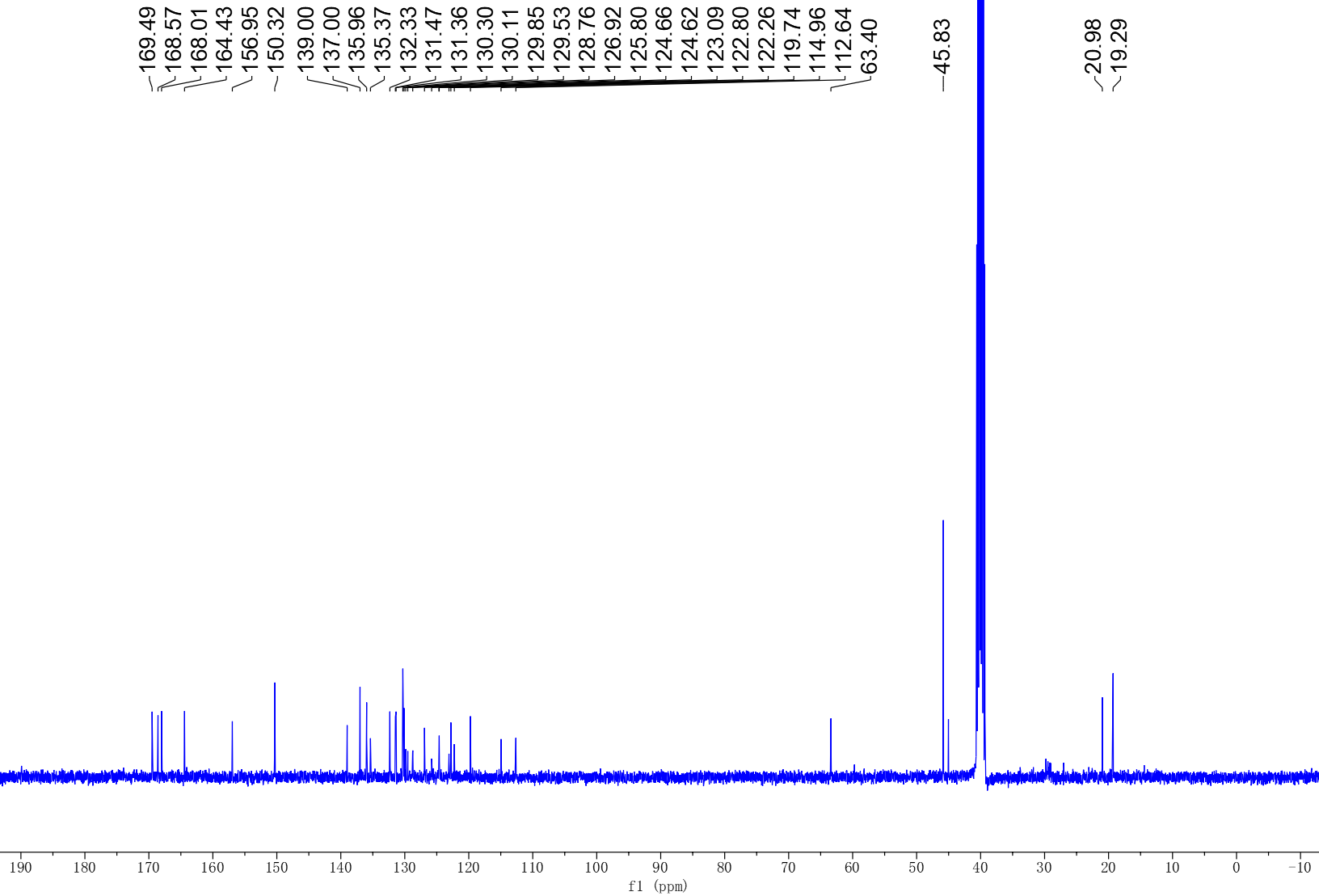


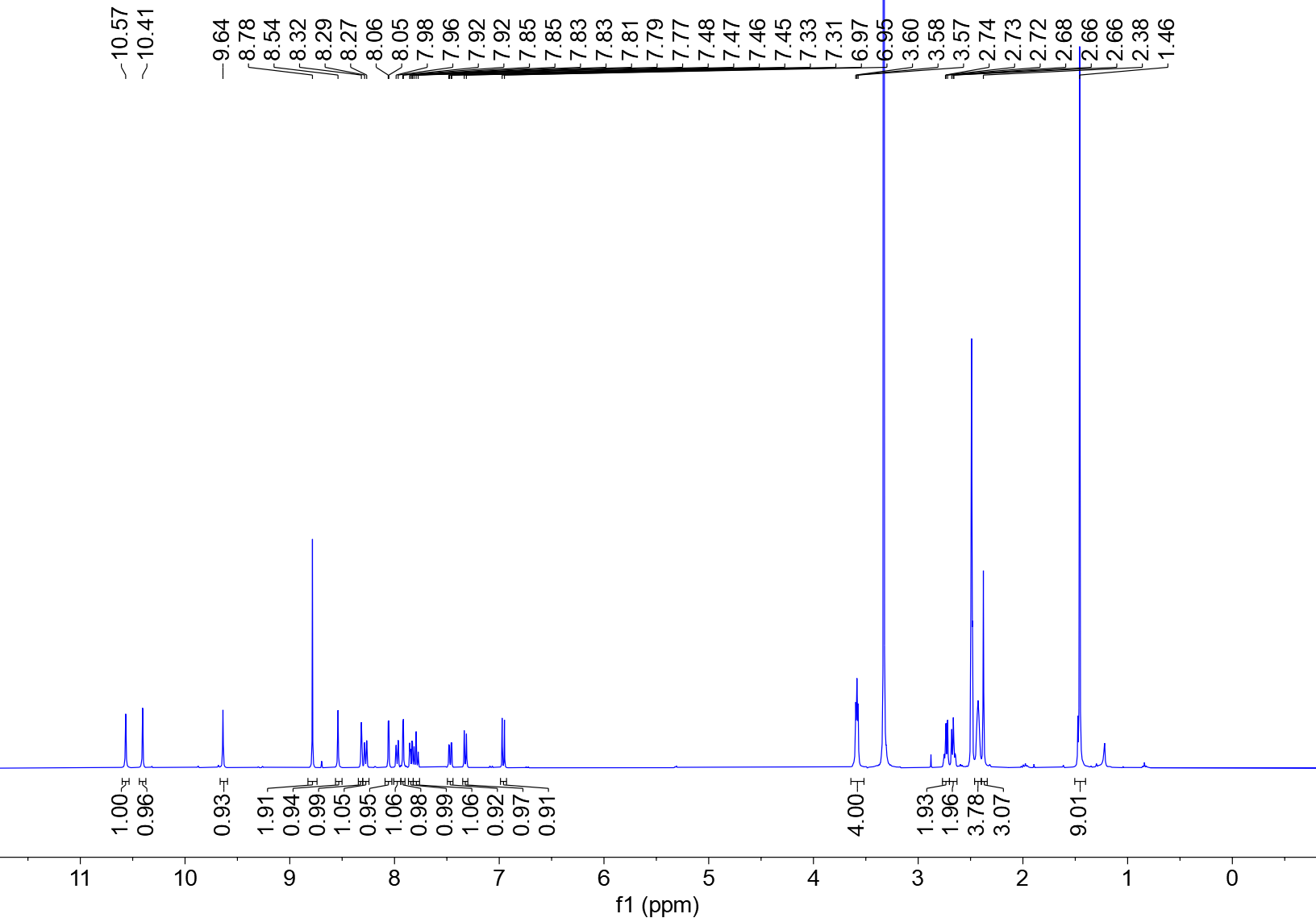


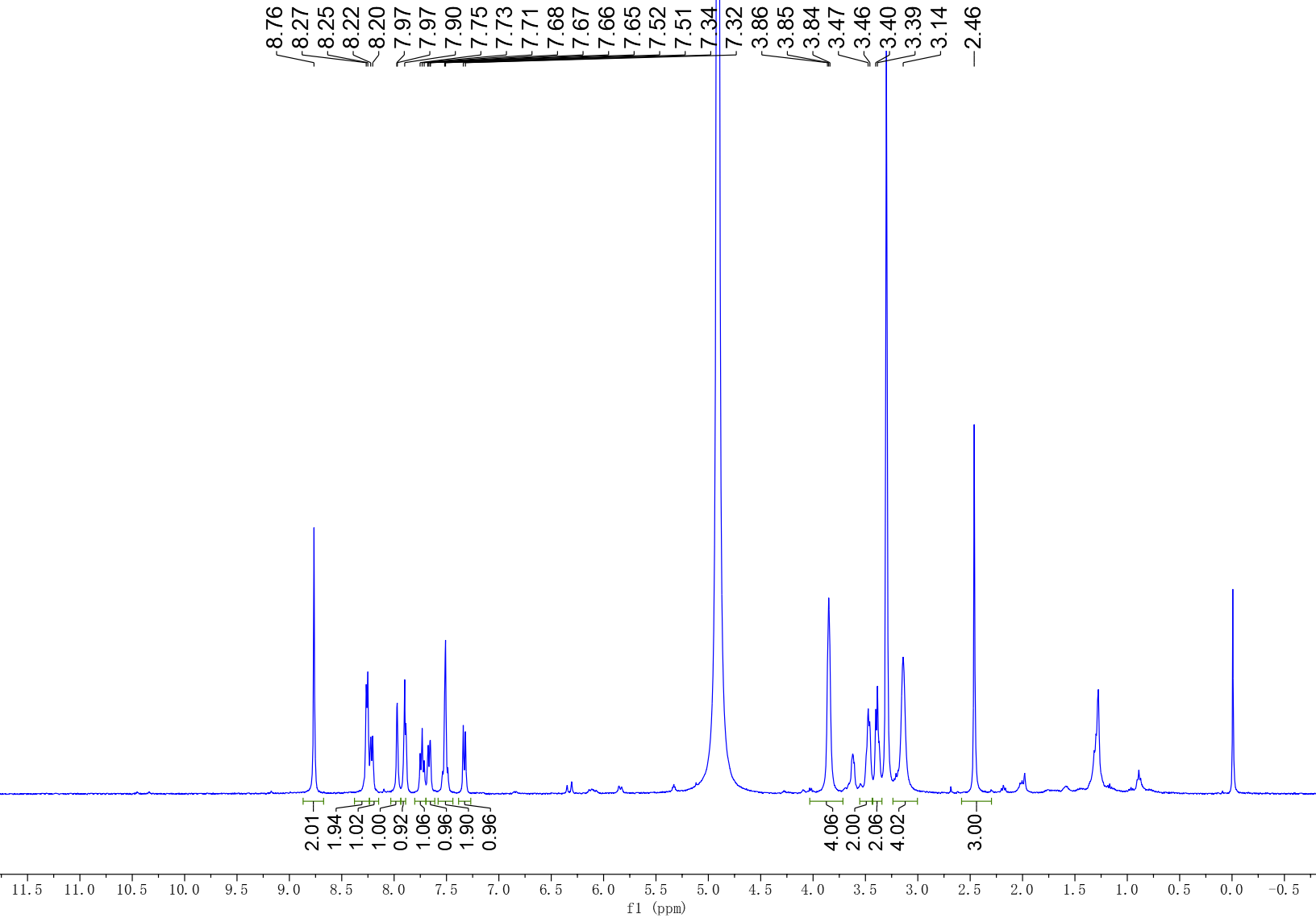


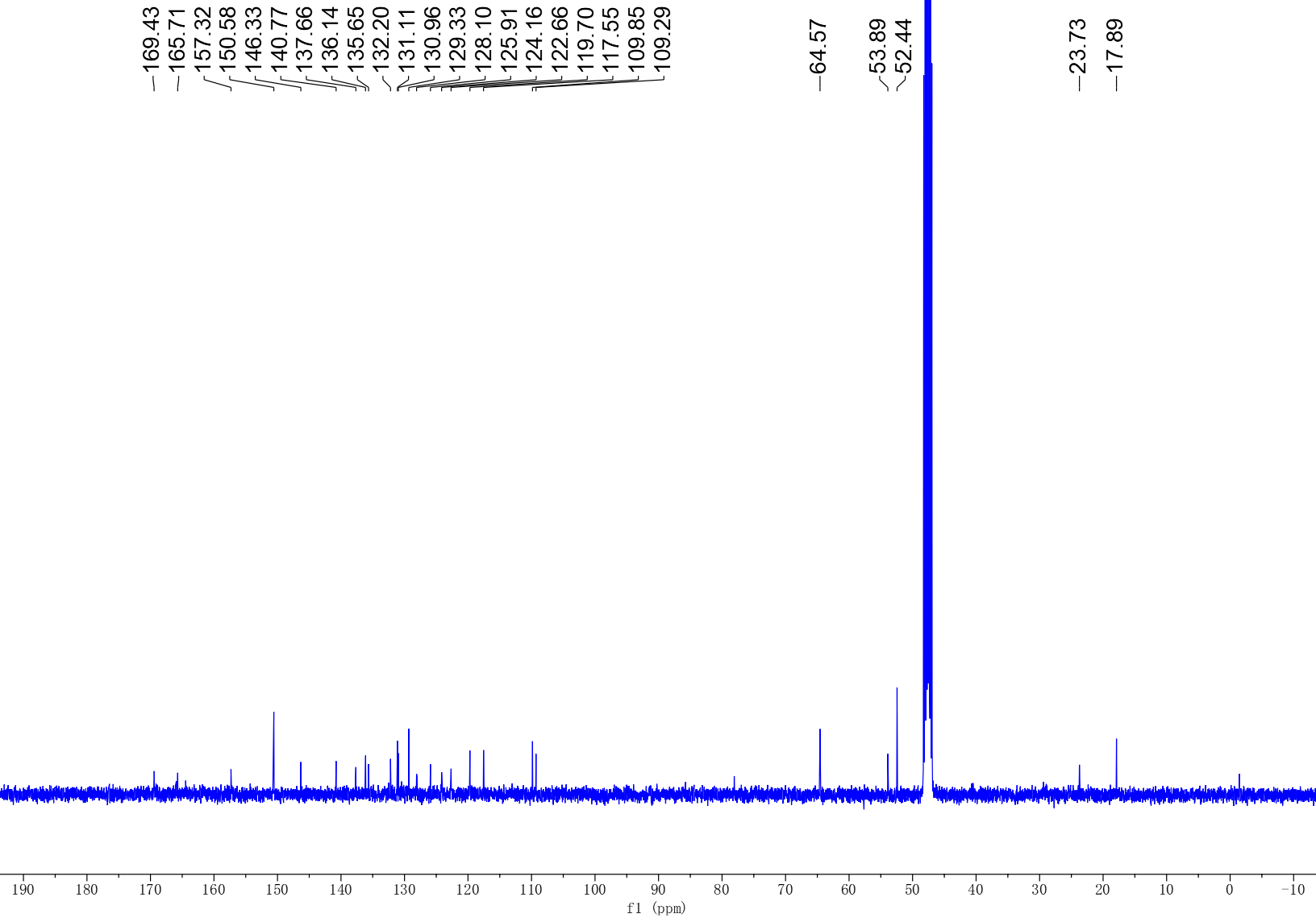


1. **Plasma stability assay**
   1. **Experimental procedures**

The in vitro stability in human plasma for the test compounds was investigated using procaine as the reference compound. The frozen human plasma was quickly thawed at 37 °C. The spiking solution was made by combining 10 µL of a 10 mM compound stock solution with 990 µL of DMSO. The plasma and spiking solution were pre-warmed at 37 ℃ for 5 min. Then 7 µL of the pre-warmed spiking solution was added into the 693 µL plasma evenly. The tests were carried out in duplicate in a 37 °C shaking water bath. At 0, 5, 15, 30, 60, and 120 min, 100 µL samples were collected and added to a 400 µL solution containing an internal standard (IS). After quenching, the samples were vortexed for 5 min (600 rpm) and then centrifuged at 4,000 rpm for 20 min. LC-MS was used to analyze the clear supernatants. The values are the mean of two separate experiments.

**Table S1. Raw data of the reference and test compounds in human plasma stability assay**

| **Compd** | Time  (min) | Raw Data | | | | | |
| --- | --- | --- | --- | --- | --- | --- | --- |
|  |  | Analyte Peak Area (counts) | Analyte Peak Area (counts) | IS Peak Area  (counts) | IS Peak Area (counts) | Area Ratio | Area Ratio |
| **Procaine** | 0 | 70100 | 69600 | 110000 | 108000 | 0.634 | 0.644 |
|  | 5 | 4570 | 4070 | 114000 | 112000 | 0.040 | 0.037 |
|  | 15 | BLOD | BLOD | 115000 | 113000 | N/A | N/A |
|  | 30 | BLOD | BLOD | 116000 | 114000 | N/A | N/A |
|  | 60 | BLOD | BLOD | 116000 | 116000 | N/A | N/A |
|  | 120 | BLOD | BLOD | 120000 | 120000 | N/A | N/A |
| **5a** | 0 | 425000 | 429000 | 150000 | 151000 | 2.830 | 2.840 |
|  | 5 | 372000 | 374000 | 152000 | 156000 | 2.450 | 2.400 |
|  | 15 | 230000 | 226000 | 159000 | 162000 | 1.440 | 1.390 |
|  | 30 | 109000 | 115000 | 156000 | 151000 | 0.699 | 0.761 |
|  | 60 | 17800 | 19300 | 159000 | 161000 | 0.112 | 0.120 |
|  | 120 | BLOD | BLOD | 164000 | 161000 | N/A | N/A |
| **4** | 0 | 159000 | 162000 | 150000 | 151000 | 1.060 | 1.080 |
|  | 5 | 274000 | 269000 | 152000 | 156000 | 1.800 | 1.730 |
|  | 15 | 595000 | 584000 | 159000 | 162000 | 3.740 | 3.600 |
|  | 30 | 863000 | 883000 | 156000 | 151000 | 5.530 | 5.830 |
|  | 60 | 1100000 | 1060000 | 159000 | 161000 | 6.920 | 6.570 |
|  | 120 | 1090000 | 1140000 | 164000 | 161000 | 6.620 | 7.110 |
| **5b** | 0 | 3050000 | 2900000 | 80600 | 75800 | 37.9 | 38.2 |
|  | 60 | 3120000 | 3170000 | 79400 | 76800 | 39.3 | 41.2 |
|  | 120 | 3060000 | 3110000 | 77900 | 77400 | 39.3 | 40.1 |

Note: BLOD: Below Limit Of Detection; N/A: Not Acquired.

1. **Molecular dynamic studies**
   1. **Experimental procedures**

Molecular dynamics simulations using the Desmond package (Schrödinger, 2018) have been carried out for the study of the conformational changes of the ligand-protein complex in the solvent environment. The docked complex simulation was run using OPLS force field parameters. The protein structure was prepared by the use of Protein Preparation Wizard in Schrödinger, and solvated in an orthorhombic box with TIP3P water molecules at least 10 Å spacing. The MD simulation was performed under an isothermal isobaric ensemble (NPT) with a pressure of 1 atm, a temperature of 300 K, and a relaxation time of the thermostat of 200 ps. The simulation was run for 100 ns with the trajectory recorded every 50 ps. The Simulation Interactions Diagram (SID) was used to analyze the MD trajectories.


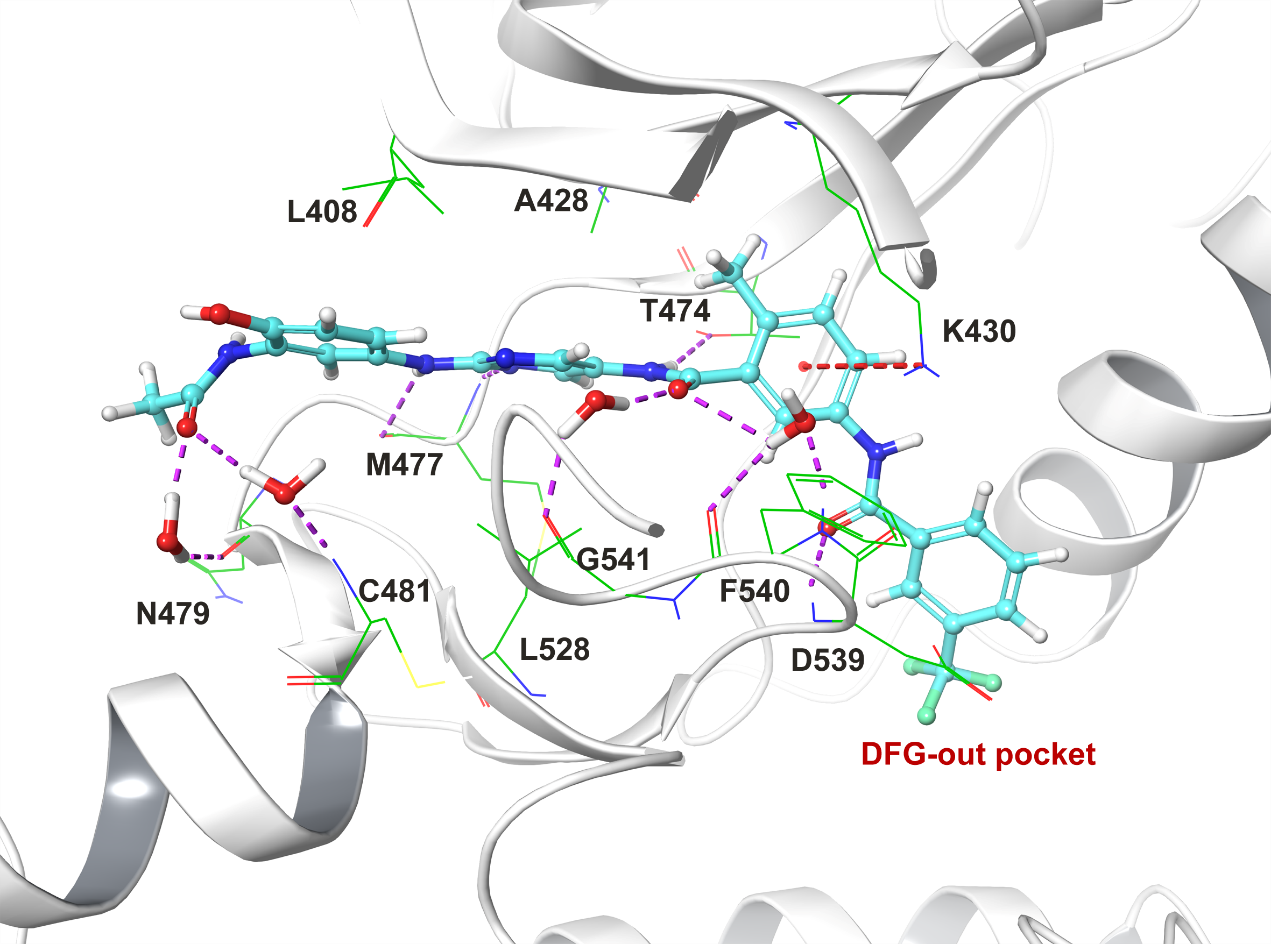


**Figure S1.** 3D view of the complex of compound **4** (carbon in cyan) in the ATP-binding pocket of BTK (PDB ID: 3pj3) after 100 ns dynamic simulation. Hydrogen bonds are visualized as dashed purple lines, π-cation interactions are visulalized as dashed red lines.
